# ﻿Three new deep-sea species of *Marphysa* (Annelida, Eunicida, Eunicidae) from Papua New Guinea (Bismarck and Solomon seas)

**DOI:** 10.3897/zookeys.1122.89990

**Published:** 2022-09-23

**Authors:** Nicolas Lavesque, Guillemine Daffe, Christopher Glasby, Stéphane Hourdez, Pat Hutchings

**Affiliations:** 1 CNRS, Université de Bordeaux, Bordeaux INP, EPOC, UMR 5805, Station Marine d’Arcachon, Arcachon, France; 2 CNRS, Université de Bordeaux, Observatoire Aquitain des Sciences de l’Univers, UMS 2567 POREA, Pessac, France; 3 Museum and Art Gallery Northern Territory, Darwin, Australia; 4 Australian Museum Research Institute, Australian Museum, 1 William Street, Sydney, NSW 2010, Australia; 5 CNRS, Sorbonne Université, Laboratoire d’Ecogéochimie des Environnements Benthiques, LECOB, Banyuls, France; 6 Department of Biological Sciences, Macquarie University, North Ryde NSW 2109, Australia

**Keywords:** Bloodworm, COI, deep sea, *Marphysa*, morphology, polychaete, sunken vegetation, taxonomy

## Abstract

Three new species of *Marphysa* Quatrefages, 1866, *Marphysabanana***sp. nov.**, *Marphysapapuaensis***sp. nov.**, and *Marphysazanolae***sp. nov.** are described from deep-sea sunken vegetation off Papua New Guinea, using both morphology and molecular data (for two species). With the presence of compound spinigers only and the branchiae present over many chaetigers, *Marphysabanana***sp. nov.** belongs to the group B2. This species is characterised by the presence of eyes, the presence of branchiae starting from chaetiger 20, and by the presence of three types of pectinate chaetae and bidentate subacicular hooks starting from chaetigers 13–52. With the presence of compound falcigers only and the branchiae restricted to a short anterior region, *Marphysapapuaensis***sp. nov.** belongs to the group C1. This species has a bilobed prostomium but no eyes, has branchiae from chaetigers 7 to 14–16 with up to 16 filaments. *Marphysapapuaensis***sp. nov.** is also characterised by the presence of bidentate subacicular hooks from chaetiger 20 and by a single type of pectinate chaetae. Finally, *Marphysazanolae***sp. nov.** belongs to the group C2, with the presence of compound falcigers only and the branchiae present over many chaetigers. This species is characterised by the absence of eyes, by the presence of branchiae with a single long filament starting from chaetiger 31, by unidentate subacicular hooks starting from chaetiger 28 and finally by one type of pectinate chaetae with very long outer teeth.

## ﻿Introduction

Situated in the Coral Triangle, Papua New Guinea is considered a marine biodiversity hotspot and shows a high level of endemism. Although the terrestrial and shallow water fauna is well known, the deep-sea fauna has rarely been studied (Pante et al. 2012). Indeed, the historical expeditions of ‘Siboga’ and the HMS ‘Challenger’, which both sampled the deep sea, did not pay much attention to this area, and only the ‘Galathea’ and RV ‘Vityaz’ expeditions carried out benthic sampling in the deep sea (hadal zone) (Pante et al. 2012; Corbari et al. 2019). Only a few taxonomic studies on polychaetes have been conducted in the region, in coastal habitats (Dahlgren 1996; Rouse 1996; Britayev et al. 1999; Rouse 2012) and hydrothermal vents (Watson 2001; Reuscher et al. 2011) but data on other types of deep-sea habitats are still lacking. Between 2010–2014, the Muséum National d’Histoire Naturelle (MNHN) and the Institut de Recherche pour le Développement (IRD), in collaboration with the University of Papua New Guinea (UPNG) launched four sampling campaigns (BIOPAPUA 2010, PAPUA NIUGINI 2012 MADEEP, and KAVIENG 2014) aiming to explore the deep-sea biodiversity of this region, especially in the Bismarck and Solomon seas (Pante et al. 2012; Corbari et al. 2019).

*Marphysa* Quatrefages, 1866 is a very speciose genus with 83 accepted species (Read and Fauchald 2022), commonly found from intertidal shores to shallow waters (Glasby and Hutchings 2010). As far as we know, and unlike *Eunice* the other species-rich genus of the family, *Marphysa* species are never found in the deep sea. Except for the non-indigenous species *Marphysavictori* Lavesque, Daffe, Bonifácio & Hutchings, 2017 (Lavesque et al. 2020), most of the species show restricted distributions (Hutchings and Kupriyanova 2018; Lavesque et al. 2019) and are often very restricted to particular habitats (Hutchings and Karageorgopoulos 2003; Glasby and Hutchings 2010; Zanol et al. 2016; Lavesque et al. 2019). Following Fauchald (1970) and Glasby and Hutchings (2010), species of the genus *Marphysa* can be separated into five artificial groups based on the type of compound chaetae: no compound chaetae present (Group A), only compound spinigers present (Group B), only compound falcigers present (Group C), both compound spinigers and falcigers present (Group D), and compound spinigers only anteriorly and posterior segments only with simple limbate chaetae (Group E). Finally, each group can also then be divided into species having branchiae present over a short anterior region (subdivision 1) or branchiae present over many chaetigers (subdivision 2).

Until now, 15 species of *Marphysa* have been described from the Central Indo-Pacific Realm (sensu Spalding et al. 2007), two species belonging to Group A (*M.fijiensis* Molina-Acevedo & Idris, 2021 and *M.moribidii* Idris, Hutchings & Arshad, 2014), nine species to Group B (*M.hongkongensa* Wang, Zhang & Qiu, 2018; *M.iloiloensis* Glasby, Mandario, Burghardt, Kupriyanova, Gunton & Hutchings, 2019; *M.maxidenticulata* Liu, Hutchings & Kupriyanova, 2018; *M.mullawa* Hutchings & Karageorgopoulos, 2003; *M.multipectinata* Liu, Hutchings & Sun, 2017; *M.orientalis* Treadwell, 1936; *M.tribranchiata* Liu, Hutchings & Sun, 2017; *M.tripectinata* Liu, Hutchings & Sun, 2017, and *M.bulla* Liu, Hutchings & Kupriyanova, 2018, which was recently synonymised with *M.victori* Lavesque, Daffe, Bonifácio & Hutchings, 2017 (Lavesque et al. 2020); two species belong to Group C (*M.bernardi* Rullier, 1972 and *M.soembaensis* Augener, 1933); and only one species belongs to each of groups D (*M.digitibranchia* Hoagland, 1920) and E (*M.fauchaldi* Glasby & Hutchings, 2010).

In this study, three new deep-sea species belonging to groups B2 (*Marphysabanana* sp. nov.), C1 (*Marphysapapuaensis* sp. nov.), and C2 (*Marphysazanolae* sp. nov.) are described using both morphology and molecular data (for two of them). The type specimens were found in deep-sea sunken vegetation (decaying wood or cultivated plants leaves). It is not surprising, as this region is known to accumulate large quantities of decomposing vegetation, transiting from tropical forests to marine canyons (Pante et al. 2012), hosting original and diverse fauna (Samadi et al. 2007).

## ﻿Materials and methods

### ﻿Sampling and morphological analyses

**Figure 1.**
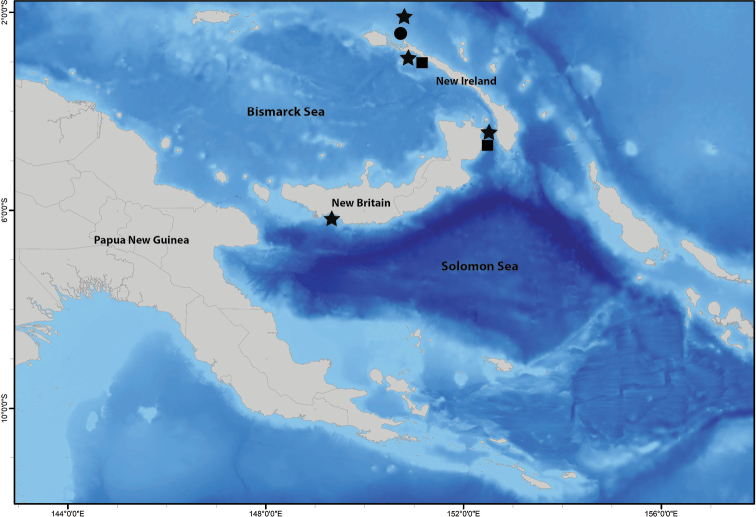
Sampling sites of type material of *Marphysabanana* sp. nov. (black circle), *Marphysapapuaensis* sp. nov. (black star), and *Marphysazanolae* sp. nov. (black square) in Papua New Guinea.

Specimens were collected by beam trawl during the MADEEP cruise (see https://expeditions.mnhn.fr/campaign/madeep) and the KAVIENG cruise (https://expeditions.mnhn.fr/campaign/kavieng2014) in May–September 2014, in the Solomon Sea (Fig. 1). All material was sorted on board RV ‘Alis’ and fixed in 80% ethanol. A few parapodia were removed from several specimens for molecular analysis. Specimens were examined under a Nikon SMZ25 stereomicroscope and a Nikon Eclipse *Ci* microscope, and photographed with a Nikon DS-Ri 2 camera. Measurements were made with the NIS-Elements Analysis software. Drawings were made from pictures using Inkscape software. Width of all specimens was obtained by measuring chaetiger 10 with parapodia. Morphological terminology is based on Fauchald (1992) for general terms, Paxton (2000) for head appendages and Molina-Acevedo and Carrera-Parra (2015) for maxillary apparatus. Terminology of pectinate chaetae follows Glasby et al. (2019), based on a previous study of Molina-Acevedo & Carrera-Parra (2017): isodont means outer teeth much longer than internal teeth, anodont means outer teeth more or less same length as internal teeth, and heterodont when one long and one short (same length as internal teeth) lateral tooth are present. The width of the pectinate blade is wide when ≥ 30 μm and narrow below this. Finally, the size of the internal teeth is long when they measure 12 µm or more and thick when are 2 µm or more, below these values the teeth are defined as short and slender, respectively.

Some parapodia along the body were removed from the type material of each species (see Material examined), dehydrated in ethanol, critical point dried, covered with 20 nm of gold, examined under the scanning electron microscope (JEOL JSM 6480LA) and imaged with a secondary detector at Macquarie University, Sydney, Australia.

The studied material is deposited at the Muséum National d’Histoire Naturelle, Paris (**MNHN**) and the Australian Museum, Sydney (**AM**). Additional material is lodged in the collection housed at the Arcachon Marine Station (**SMA**).

### ﻿Molecular data and analyses

Extraction of DNA was done with ISOLATE II Genomic DNA kit (BIOLINE) following protocol supplied by the manufacturers. Approximately 600 bp of COI (cytochrome c oxidase subunit I) gene was amplified, using primers polyLCO and polyHCO COI (Carr et al. 2011). PCR (Polymerase Chain Reaction) was performed with Taq DNA Polymerase QIAGEN Kit in 20 μL mixtures containing: 2 μL of 10X CoralLoad PCR Buffer (final concentration of 1X), 1.5 μL of MgCl2 (25 Mm) solution, 1.5 μL of PCR nucleotide mix (final concentration of 0.2 mM each dNTP), 0.4 μl of each primer (final concentration of 0.2 μM), 0.1 μl of Taq DNA Polymerase (5U/μl), 1 μl template DNA and 13.1 μL of nuclease-free water. The temperature profile was as follows 94 °C / 60 s – (94 °C / 40 s – 45 °C / 40 s – 72 °C / 60 s)*5 cycles – (94 °C / 40 s – 51 °C/ 40 s – 72 °C / 60 s)*35 cycles – 72 °C / 300 s – 4 °C. PCR success was verified by electrophoresis in a 1% p/v agarose gel stained with Gelred. Amplified products were sent to Macrogen Company to obtain sequences, using the same set of primers as used for PCR.

Fifty-nine COI sequences were downloaded from GenBank or obtained during this study, fifty-six COI sequences of *Marphysa* species and three outgroup species from closely related genera in the family Eunicidae (Table 1). During this study, one COI sequence was obtained both for *Marphysapapuaensis* sp. nov. and *Marphysazanolae* sp. nov., but we failed to obtain a sequence for *Marphysabanana* sp. nov.

**Table 1.** Terminal taxa used in the molecular part of the study (COI gene), with type localities, collection localities, GenBank accession numbers, and references.

Species	Type locality	Collection locality	GenBank accession number	Reference
Eunicecf.violaceomaculata	Tortugas, Caribbean	Carrie Bow Cay, Belize	GQ497542	Zanol et al. 2010
*Palolaviridis*	Samoa, Pacific Ocean	Kosrae, Micronesia	GQ497556	Zanol et al. 2010
*Leodicerubra*	Saint Thomas, Caribbean	Ceara, Brazil	GQ497528	Zanol et al. 2010
*M.aegypti*	Suez Canal, Egypt	Suez Canal, Egypt	MF196969	Elgetany et al. 2018
*M.bifurcata*	WA, Australia	Qld, Australia	KX172177	Zanol et al. 2016
*M.bifurcata*	WA, Australia	Qld, Australia	KX172178	Zanol et al. 2016
*M.brevitentaculata*	Tobago	Quintana Roo, Mexico	GQ497548	Zanol et al. 2010
*M.californica*	California, USA	California, USA	GQ497552	Zanol et al. 2010
*M.disjuncta*	California, USA	California, USA	GQ497549	Zanol et al. 2010
*M.chirigota*	Bay of Cadiz, Spain	Bay of Cadiz, Spain	MN816442	Martin et al. 2020
*M.chirigota*	Bay of Cadiz, Spain	Bay of Cadiz, Spain	MN816443	Martin et al. 2020
*M.chirigota*	Bay of Cadiz, Spain	Bay of Cadiz, Spain	MN816444	Martin et al. 2020
*M.fauchaldi*	NT, Australia	NT, Australia	KX172165	Zanol et al. 2016
*M.gaditana*	Bay of Cadiz, Spain	Bay of Cadiz, Spain	MN816441	Martin et al. 2020
*M.hongkongensa*	Hong Kong	Hong Kong	MH598525	Wang et al. 2018
*M.hongkongensa*	Hong Kong	Hong Kong	MH598526	Wang et al. 2018
*M.iloiloensis*	Iloilo, Philippines	Tigbauan, Philippines	MN106279	Glasby et al. 2019
*M.iloiloensis*	Iloilo, Philippines	Tigbauan, Philippines	MN106280	Glasby et al. 2019
*M.iloiloensis*	Iloilo, Philippines	Tigbauan, Philippines	MN106281	Glasby et al. 2019
*M.kristiani*	NSW, Australia	NSW, Australia	KX172160	Zanol et al. 2016
*M.kristiani*	NSW, Australia	NSW, Australia	KX172161	Zanol et al. 2016
*M.kristiani*	NSW, Australia	NSW, Australia	KX172162	Zanol et al. 2016
*M.kristiani*	NSW, Australia	NSW, Australia	KX172158	Zanol et al. 2016
*M.madrasi*	Chennai, India	Chennai, India	MT813506	Hutchings et al. 2020
*M.madrasi*	Chennai, India	Chennai, India	MT813507	Hutchings et al. 2020
*M.mossambica*	Mozambique	Iloilo, Philippines	KX172164	Zanol et al. 2016
*M.mullawa*	Qld, Australia	NSW, Australia	KX172166	Zanol et al. 2016
*M.mullawa*	Qld, Australia	NSW, Australia	KX172167	Zanol et al. 2016
*M.mullawa*	Qld, Australia	NSW, Australia	KX172168	Zanol et al. 2016
*M.mullawa*	Qld, Australia	NSW, Australia	KX172176	Zanol et al. 2016
*M.papuaensis* sp. nov.	Papua New Guinea	Papua New Guinea	OP184050	This study
*M.pseudosessiloa*	NSW, Australia	NSW, Australia	KY605405	Zanol et al. 2010
*M.pseudosessiloa*	NSW, Australia	NSW, Australia	KY605406	Zanol et al. 2010
*M.regalis*	Bermuda	Ceara, Brazil	GQ497562	Zanol et al. 2010
*M.sanguinea*	Devon, UK	Callot Island, France	GQ497547	Zanol et al. 2010
*M.sanguinea*	Devon, UK	Cornwall, UK	MK541904	Lavesque et al. 2019
*M.sanguinea*	Devon, UK	Arcachon Bay, France	MK950853	Lavesque et al. 2019
*M.sanguinea*	Devon, UK	Brest, France	MK967470	Lavesque et al. 2019
*M.tripectinata*	Beihai, China	Beihai, China	MN106271	Glasby et al. 2019
*M.sherlockae*	Durban, South Africa	Strand, South Africa	MT840349	Kara et al. 2020
*M.sherlockae*	Durban, South Africa	Strand, South Africa	MT840350	Kara et al. 2020
*M.sherlockae*	Durban, South Africa	Strand, South Africa	MT840351	Kara et al. 2020
*M.tripectinata*	Beihai, China	Beihai, China	MN106272	Glasby et al. 2019
*M.tripectinata*	Beihai, China	Beihai, China	MN106273	Glasby et al. 2019
*M.tripectinata*	Beihai, China	Beihai, China	MN106274	Glasby et al. 2019
*M.tripectinata*	Beihai, China	Beihai, China	MN106275	Glasby et al. 2019
*M.tripectinata*	Beihai, China	Beihai, China	MN106276	Glasby et al. 2019
*M.tripectinata*	Beihai, China	Beihai, China	MN106277	Glasby et al. 2019
*M.tripectinata*	Beihai, China	Beihai, China	MN106278	Glasby et al. 2019
*M.victori*	Arcachon Bay, France	Arcachon Bay, France	MG384996	Lavesque et al. 2017
*M.victori*	Arcachon Bay, France	Arcachon Bay, France	MG384997	Lavesque et al. 2017
*M.victori*	Arcachon Bay, France	Arcachon Bay, France	MG384998	Lavesque et al. 2017
*M.victori*	Arcachon Bay, France	Arcachon Bay, France	MG384999	Lavesque et al. 2017
*M.victori*	Arcachon Bay, France	Mangoku-ura Inlet, Japan	LC467767	Abe et al. 2019
*M.victori*	Arcachon Bay, France	Sendai Bay, Japan	LC467769	Abe et al. 2019
*M.victori*	Arcachon Bay, France	Ena Bay, Japan	LC467772	Abe et al. 2019
*M.victori*	Arcachon Bay, France	China	MT012514	Lavesque et al. 2020
*M.viridis*	Florida, USA	Ceara, Brazil	GQ497553	Zanol et al. 2010
*M.zanolae* sp. nov.	Papua New Guinea	Papua New Guinea	OP184049	This study

All COI sequences were aligned in Geneious Prime 2019.0.4 using the MUSCLE plugin and default settings. The AIC and BIC tests in jModeltest 2.2.10 (Darriba et al. 2012) were used to select the GTR + I + G model of molecular evolution as the best evolutionary model for the COI gene alignment. The phylogenetic analysis was performed in MrBayes v. 3.2.6 (Ronquist and Huelsenbeck 2003). The analysis was run for 10 million generations (sampled every 1000), 25% of the generations were discarded as burn-in and the standard deviation of split frequencies decreased below 0.01. FigTree v. 1.4.4 (Rambaut 2007) was used to visualise the majority-rule consensus tree displaying all nodes with a posterior probability > 0.5. Pair-wise Kimura 2-parameter (K2P) genetic distance was performed using MEGA v. 7.0.26.

## ﻿Taxonomic account

### ﻿Family Eunicidae Berthold, 1827

#### ﻿Genus Marphysa

Taxon classificationAnimaliaEunicidaEunicidae

Quatrefages, 1866

95959A1E-FC26-545C-96F3-9DE2447356BB

##### Type species.

*Nereissanguinea* Montagu, 1813.

#### ﻿ Marphysa banana sp. nov.

Taxon classificationAnimaliaEunicidaEunicidae

A6A8C2F0-7DC4-5B8B-BBC9-6ED82DA82C0B

https://zoobank.org/36B09BD6-2080-4266-9945-98EA1CA40913

Figs 2 , 3 , 4

##### Material examined.

***Holotype***: MNHN-IA-2015-1608, complete. ***Paratypes***: AM W.53773, complete; AM W.53774, complete, some parapodia mounted for SEM; MNHN-IA-2021-725, anterior part only. All material collected from South Pacific Ocean, Papua New Guinea, New Ireland, CP4254, -2.483°S, 150.66°E, depth 273–324 m, April 2014.

##### Description

**Figure 2.**
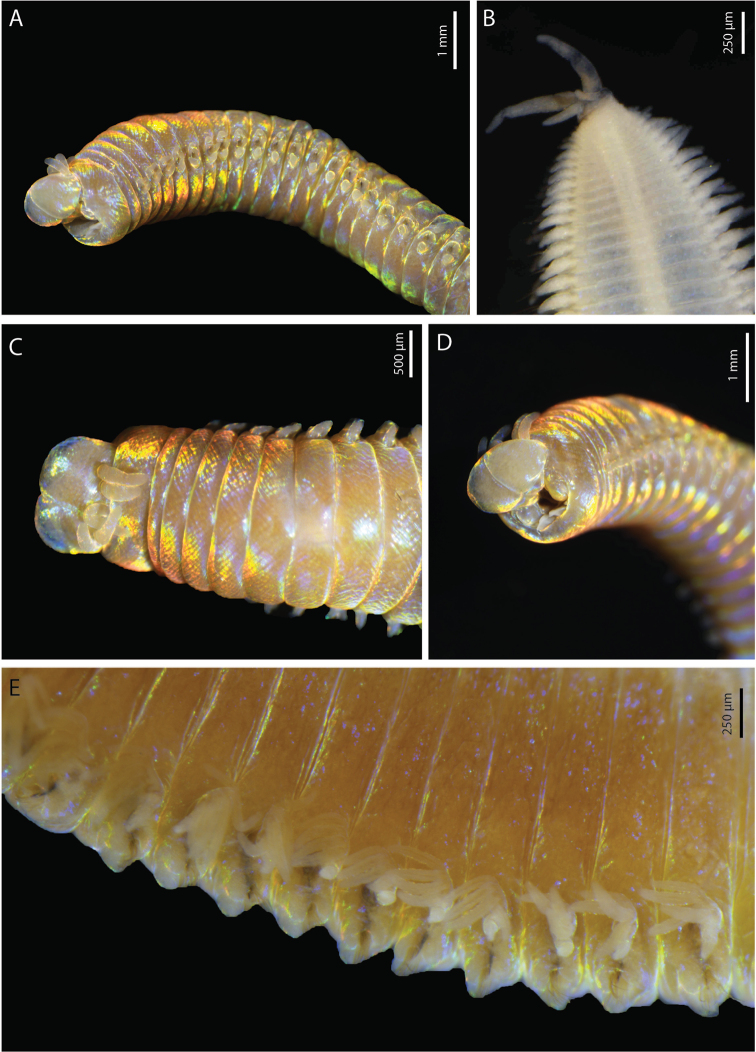
*Marphysabanana* sp. nov. holotype MNHN-IA-2015-1608 (**B**) paratype MNHN-IA-2021-725 (**A, C–E**): **A** anterior end, lateral view **B** pygidium, ventral view **C** anterior end, dorsal view **D** anterior end, frontal view **E** anterior chaetigers, dorso-lateral view.

(based on holotype, with variation in parentheses for paratypes). Preserved specimens strongly iridescent (Fig. 2A, C, D), ~ 230 (220) chaetigers, 112 mm (71–157) long, 3.3 mm (2.1–5.4) width at chaetiger 10, excluding parapodia. Body elongated and tapered gradually at posterior end (Fig. 2B).

Prostomium rounded anteriorly with two dorsoventrally flattened buccal lips an anterior notch between them, notch more visible ventrally (Fig. 2A, C, D). Two palps and three antennae slender and tapering, arranged in an arc on posterior margin of prostomium. Antennae more or less smooth, of equal length, longer than palps (same size), shorter (same size) than prostomium (Fig. 2C). Eyes present, one pair, brownish, very faint, present at posterior base between palps and lateral antennae. First peristomial ring ~ 3× longer than second one dorsally (Fig. 2C).

**Figure 3.**
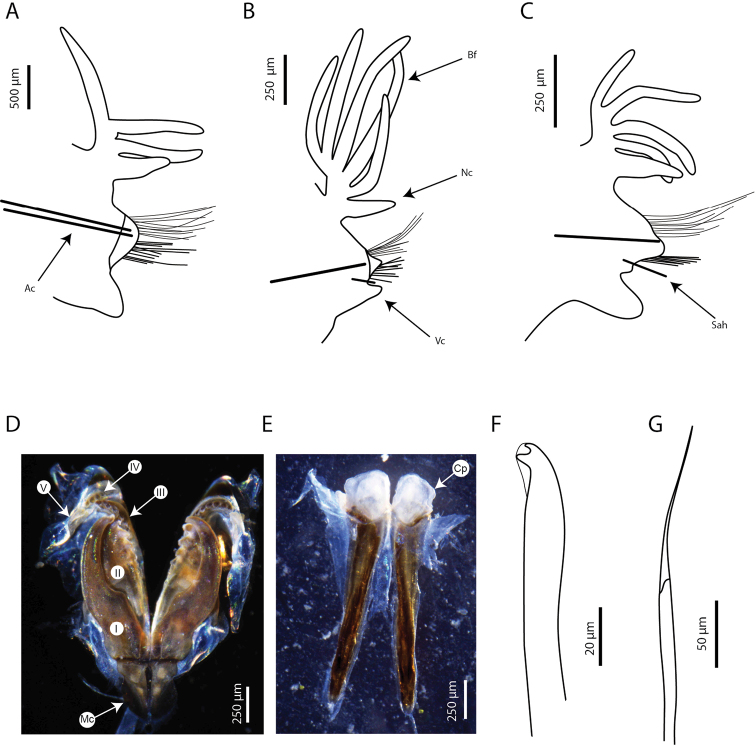
*Marphysabanana* sp. nov. holotype MNHN-IA-2015-1608 (**A–C, F–G**), paratype MNHN- IA-2021-725 (**D, E**): **A** parapodia from anterior chaetiger (chaetiger 29) **B** parapodia from mid-body (chaetiger 100) **C** parapodia from posterior chaetiger (chaetiger 190) **D** maxilla, dorsal view **E** mandibles, dorsal view **F** subacicular hook from mid-body (chaetiger 100) **G** spiniger from chaetiger 29. Abbreviations: MI to MV, maxillae I to V; Ac, aciculae; Bf, branchial filament; Cp, cutting plate; Mc, maxillary carrier; Nc, notopodial cirri; Sah, subacicular hook; Vc, ventral cirri. **A–C** Sah and Ac are illustrated schematically to indicate position.

Maxillary apparatus (Fig. 3D, E) partially everted in holotype or paratypes. Formula as follows: MF = 1+1, 5+5, 6+0, 4+9, 1+1. MI ~ 2× longer than maxillary carrier, rectangular anteriorly, triangular posteriorly, with a pair of rounded wings situated at posterolateral margins. MI forceps-like, without attachment lamellae, sub-right-angle falcal arch. Closing system ~ 4× shorter than MI. Ligament between MI and MII golden. MII without attachment lamella, teeth triangular, distributed in less than half of plate length. Ligament between MII and MIII absent (or not sclerotized). MIII, single, longer than left MIV, curved, with equal-sized triangular teeth; short attachment lamella situated in the centre of posterior edge of maxilla, thin, dark. Left MIV short (less than half the size of right MIV) with wide, rounded base, left-most teeth longer than right-most ones; attachment lamella dark, semi-circular. Right MIV long, with teeth triangular, decreasing in size posteriorly; attachment lamella wide, semi-circular, dark. MV, paired, as long as high, with a dorsal curved tooth. Mandibles light brown, concentric stripes not visible; longer than MI; cutting plates whitish, without dorsal teeth (Fig. 3E).

First few parapodia located below middle line of body wall, but gradually positioned dorsally to approximately midline in subsequent segments (Fig. 2A). Notopodial cirri slender, tapering, slightly longer than ventral cirri, thinner posteriorly (Fig. 3A–C). Chaetal lobes comprising a low pre-chaetal lip and a globular post-chaetal lobe. Ventral cirri bluntly conical, with rounded tip, shorter than post-chaetal lobes anteriorly, thereafter slightly longer than post-chaetal lobes (Fig. 3A–C). Branchiae pectinate, commencing from chaetiger 20 (18–19) and continuing to near end, very short anteriorly, longer in medium chaetigers but not reaching mid-dorsal line; number of filaments increasing from 1–3 anteriorly to 4–6 in mid-body, decreasing to 3–4 in last several chaetigers (Figs 2E, 3A–C).

Aciculae black with paler blunt tips, approximately three or four per parapodium in anterior chaetigers, one or two per parapodium in middle chaetigers, and one per parapodium in posterior chaetigers. Supra-acicular chaetae with limbate capillaries and pectinates; capillaries present from first chaetiger to near pygidium, numbering up to 20 in anterior chaetigers (Fig. 3A–C).

**Figure 4.**
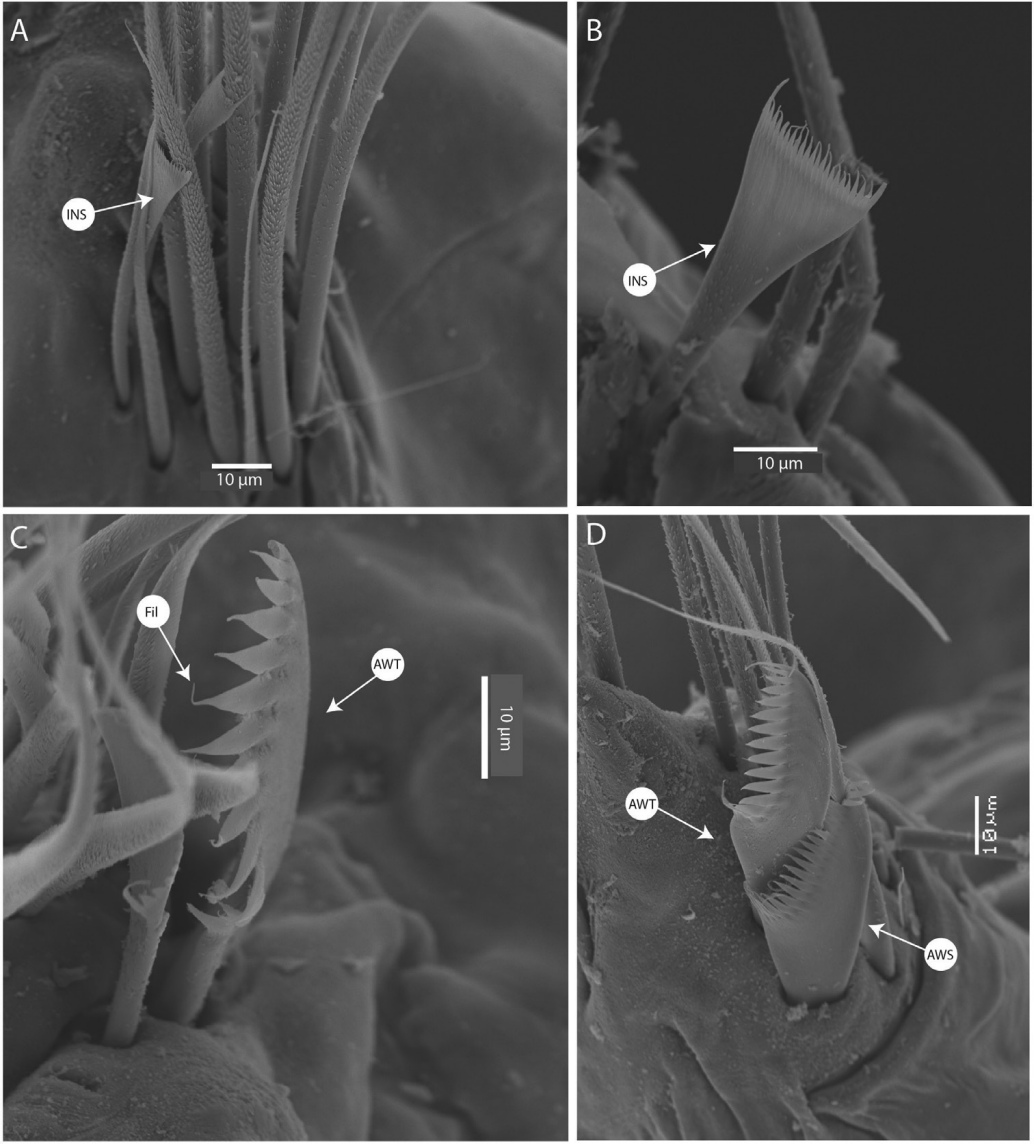
SEM images of pectinate chaetae of *Marphysabanana* sp. nov. paratype AM W.53774 **A** chaetiger 35 **B** chaetiger 67 **C** chaetiger 99 **D** chaetiger 131. Abbreviations: AWS, anodont-wide-slender; AWT, anodont-wide-thick; INS, isodont-narrow-slender; Fil, filament.

Pectinate chaetae commencing from approximately chaetiger 20 to near end, three types identified. Type 1 from anterior parapodia to mid-body: isodont-narrow-slender (INS), having ~ 20 short internal teeth, each tooth prolonged by a thin filament (Fig. 4A, B). Types 2 and 3 from posterior parapodia only (Fig. 4C, D): type 2 asymmetrical, anodont-wide-thick (AWT), having ~ 10 thick internal teeth, each tooth prolonged by a thin filament; type 3 asymmetrical, anodont-wide-slender (AWS), having ~ 20 internal teeth, each tooth prolonged by a thin filament (Fig. 4D).

Subacicular chaetae with compound spinigers and subacicular hooks (Fig. 3F). Compound spinigers commencing from first chaetiger to near pygidium, with long, tapered blade. Subacicular hooks transparent, commencing from anterior chaetigers 43–52 (range for type material) to near end and inferior to bundle of spinigers, one per parapodium; much thinner than aciculae; subacicular hooks bidentate (Fig. 3F).

Pygidium round, with crenulated margin, dorsally positioned, with two pairs of tapering pygidial cirri attached at ventral edge, dorsal pair 2–3× length of ventral pair (Fig. 2B).

##### Etymology.

The species name refers to the decomposing banana leaves among which all the specimens were found.

##### Type locality.

South Pacific, Papua New Guinea, New Ireland.

##### Distribution.

Only known from type locality.

##### Habitat.

Between 273 and 324 m, found inside banana leaves that presumably have been entrained from river runoff via coastal waters.

##### Remarks.

With the presence of compound spinigers only and the branchiae present over many chaetigers *Marphysabanana* sp. nov. belongs to the group B2, also known as the *sanguinea*-group Quatrefages, 1866. Among the nine species of this group occurring in the Central Indo-Pacific Realm, *M.banana* sp. nov. is similar to *M.hongkongensa*, *M.iloiloensis*, and *M.mullawa* by the presence of subacicular hooks starting from chaetigers 30–50 and the branchiae commencing from chaetigers 14–20.

However, *M.banana* sp. nov. differs from *M.hongkongensa* by the presence of pectinate chaetae starting from around chaetiger 20 instead of starting from the first few chaetigers as found for *M.hongkongensa*; and by the presence of three different types of pectinate chaetae instead of four types as found in *M.hongkongensa*. Moreover, *M.banana* sp. nov. has eyes whereas *M.hongkongensa* does not have any. The subacicular hooks of *M.banana* sp. nov. are bidentate while those of *M.hongkongensa* are unidentate and the maximum number of branchial filaments reaches six for *M.banana* sp. nov., while it can be ten for *M.hongkongensa*. Finally, *M.hongkongensa* lives in the lower intertidal of the Hong Kong region, while *M.banana* sp. nov. is a deep-sea species found inside banana leaves.

*Marphysabanana* sp. nov. differs from *M.iloiloensis* by the presence of four and nine teeth on the maxillary MIV, while *M.iloiloensis* has three and five teeth respectively. The two species show three different types of pectinate chaetae but not the same ones, as *M.banana* sp. nov. has INS, AWT and AWS with the first ones starting from chaetiger 20 while *M.iloiloensis* has INS, IWS and ANT, with first ones commencing from the first few chaetigers. The subacicular hooks are also different as they are bidentate for *M.banana* sp. nov. and unidentate for *M.iloiloensis*. Finally, *M.iloiloensis* lives in the brackish waters of the Philippines region, which is a very different habitat from the deep-sea habitat of *M.banana* sp. nov.

*Marphysabanana* sp. nov. differs from *M.mullawa* by the presence of pectinate chaetae starting from around chaetiger 20 instead of commencing from the first few chaetigers for *M.mullawa*, and the anterior chaetae numbering ~ 20 internal teeth instead of 10 for *M.mullawa*. The two species differ by their maxillary formulae, especially for maxillary MII (7+7 for *M.mullawa*, 5+5 for *M.banana* sp. nov.) and MIII (8+0 for *M.mullawa*, 6+0 for *M.banana* sp. nov.). Another difference concerns the shape of anterior branchiae, which are palmate for *M.mullawa* and pectinate for *M.banana* sp. nov. Once again, the two species live in very different habitats as *M.mullawa* is found in intertidal and shallow waters only, on mud or in seagrass beds.

#### ﻿ Marphysa papuaensis sp. nov.

Taxon classificationAnimaliaEunicidaEunicidae

EF15540A-E13B-5319-983A-EBD0E579298D

https://zoobank.org/991E2117-4921-4A7F-83B8-8F30EF6CA73C

Figs 5 , 6 , 7

##### Material examined.

***Holotype***: MNHN-IA-2015-1559, complete, South Pacific Ocean, Papua New Guinea, New Britain, CP4264, -4.6°S, 152.4°E, depth 430–523 m, April 2014. ***Paratypes***: MNHN-IA-2015-1415, complete, South Pacific Ocean, Papua New Guinea, New Britain, CP4337, -6.083°S, 149.316°E, depth 287–447 m, May 2014; MNHN-IA-2015-1593, anterior part only, South Pacific Ocean, Papua New Guinea, New Britain, CP4329, -6.133°S, 149.166°E, depth 250–500 m, May 2014; AM W.53770, complete (several parapodia mounted for SEM), South Pacific Ocean, Papua New Guinea, New Britain, CP4264, 4.6°S, 152.4°E, depth 430–523 m, April 2014; AM W.53771, anterior part only, mounted for SEM, South Pacific Ocean, Papua New Guinea, New Britain, CP4334, -6.116°S, 149.166°E, depth 430–620 m, May 2014; AM W.53772, complete, gravid, South Pacific Ocean, Papua New Guinea, New Britain, CP4266, -4.6166°S, 152.416°E, depth 575–616 m, April 2014.

##### Additional material.

MNHN-IA-2015-1610, anterior part only, gravid, South Pacific Ocean, Papua New Guinea, New Ireland, CP4260, -2.9°S, 151.1°E, depth 350–847 m, April 2014; MNHN-IA-2015-1949, anterior part only, South Pacific Ocean, Papua New Guinea, New Ireland, CP4434, -2.25°S, 150.8°E, depth 1066–1200 m, August 2014; MNHN-IA-2015-1615, anterior part only, few parapodia used for molecular analysis, South Pacific Ocean, Papua New Guinea, New Hanover, CP4482, -2.683°S, 150.116°E, depth 761–825 m, September 2014.

##### Description

**Figure 5.**
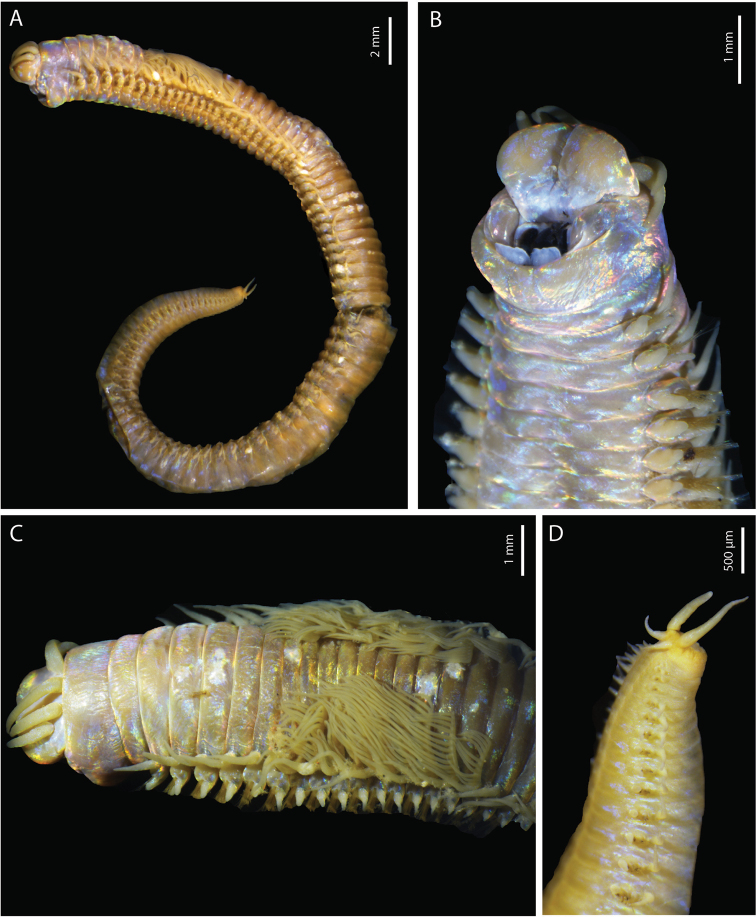
*Marphysapapuaensis* sp. nov. holotype MNHN-IA-2015-1559: **A** entire specimen, lateral view **B** anterior end, ventral view **C** anterior end, dorsal view **D** pygidium, lateral view.

(based on holotype, with variation in parentheses for paratypes). Specimens strongly iridescent (Fig. 5B), 88 (89) chaetigers, 45 mm (41–80) long, 3.6 mm (2.5–2.8) width at chaetiger 10, excluding parapodia. Body elongated and tapered gradually at posterior end, anteriorly not flattened (Fig. 5A).

Prostomium bilobed, with buccal lips separated by a ventral notch only (Fig. 5B). Two palps and three antennae slender and tapering, palpophores not visible, arranged in an arc on posterior margin of prostomium. Antennae more or less smooth, of equal length, slightly longer than palps and prostomium (same size) (Figs 5C, 7A). Eyes absent. First peristomial ring ~ 1.8× longer than second one dorsally (Figs 5C, 7A).

**Figure 6.**
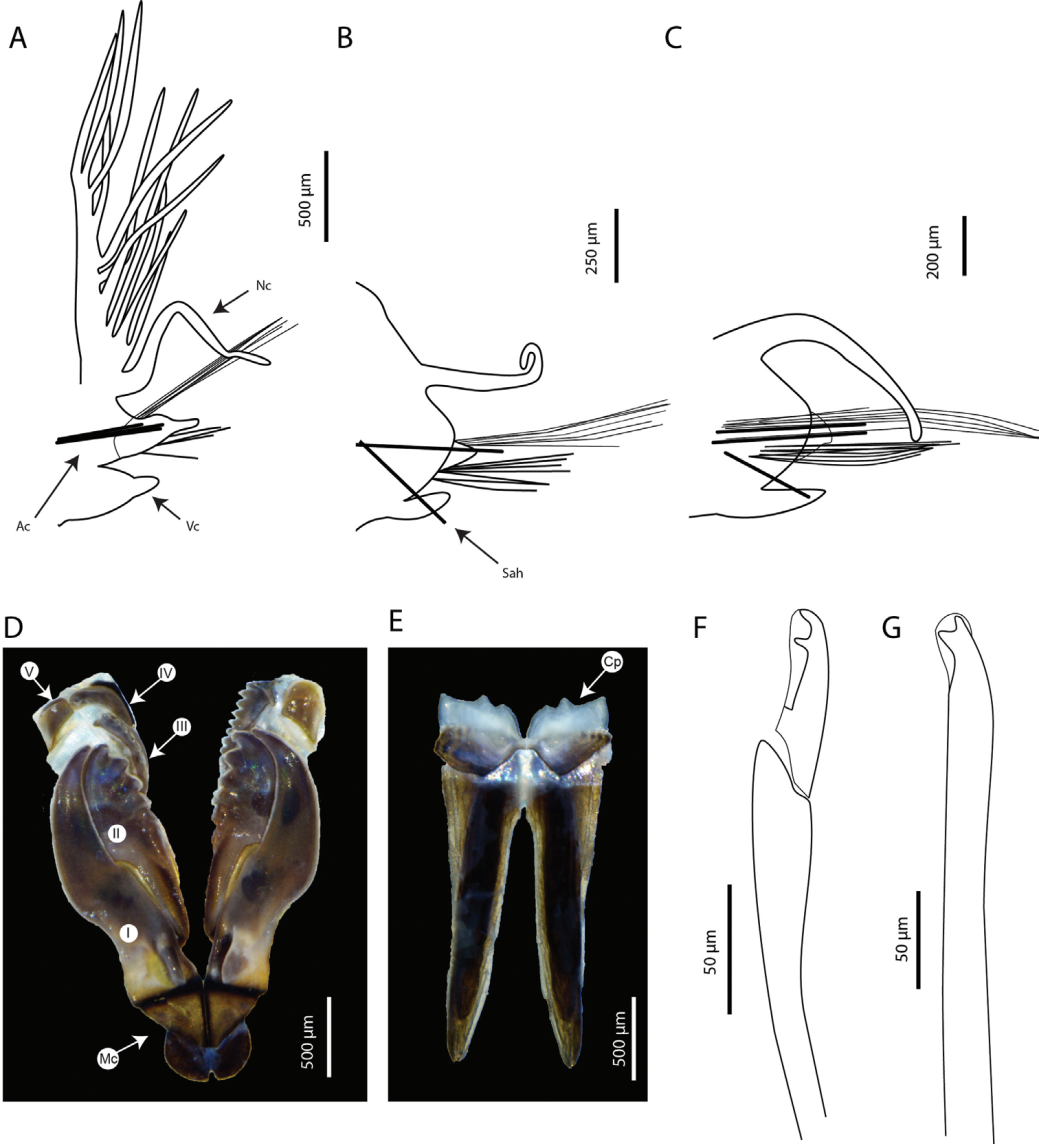
*Marphysapapuaensis* sp. nov. paratypes MNHN-IA-2015-1415 (**A–C, F, G**), MNHN-IA-2015-1593 (**D, E**): **A** parapodia from anterior chaetiger (chaetiger 12) **B** parapodia from mid-body (chaetiger 36) **C** parapodia from posterior chaetiger (chaetiger 74) **D** maxilla, dorsal view **E** mandibles, dorsal view **F** compound falcigers from anterior chaetiger (chaetiger 12) **G** subacicular hook from mid-body (chaetiger 36). Abbreviations: MI to MV, maxillae I to V; Ac, aciculae; Cp, cutting plate; Mc, maxillary carrier; Nc, notopodial cirri; Sah, subacicular hook; Vc, ventral cirri. **A–C** Sah and Ac are illustrated schematically to indicate position.

Maxillary apparatus (Fig. 6D, E) partially everted in holotype or paratypes. Formula as follows: MF = 1+1, 5(6)+5(6), 7+0, 4(3)+10(11), 1+1. MI ~ 2.5× longer than maxillary carrier, rectangular anteriorly, triangular posteriorly, with a pair of rounded wings situated at posterolateral margins. MI forceps-like, without attachment lamellae, sub-right-angle falcal arch. Closing system ~ 4–5× shorter than MI. Ligament between MI and MII rectangular, dark. MII without attachment lamella, teeth triangular, distributed in less than half of plate length. Ligament between MII and MIII absent (or not sclerotized). MIII, single, longer than left MIV, curved, with equal-sized triangular teeth; short attachment lamella situated in the centre of posterior edge of maxilla, oval, dark. Left MIV short (half the size of right MIV) with wide, rounded base, two left teeth longer than right-most ones; attachment lamella dark, semi-circular. Right MIV long, with teeth triangular, decreasing in size posteriorly; attachment lamella wide, semi-circular, dark. MV, paired, longer than wide, with a long tooth pointed ventrally, and a rounded dorsal margin (Fig. 6D). Mandibles dark with golden tips, with fine concentric stripes visible dorsally and ventrally, same size as MI; cutting plates whitish, with distinct growth rings, with three dorsal teeth (Fig. 6E).

**Figure 7.**
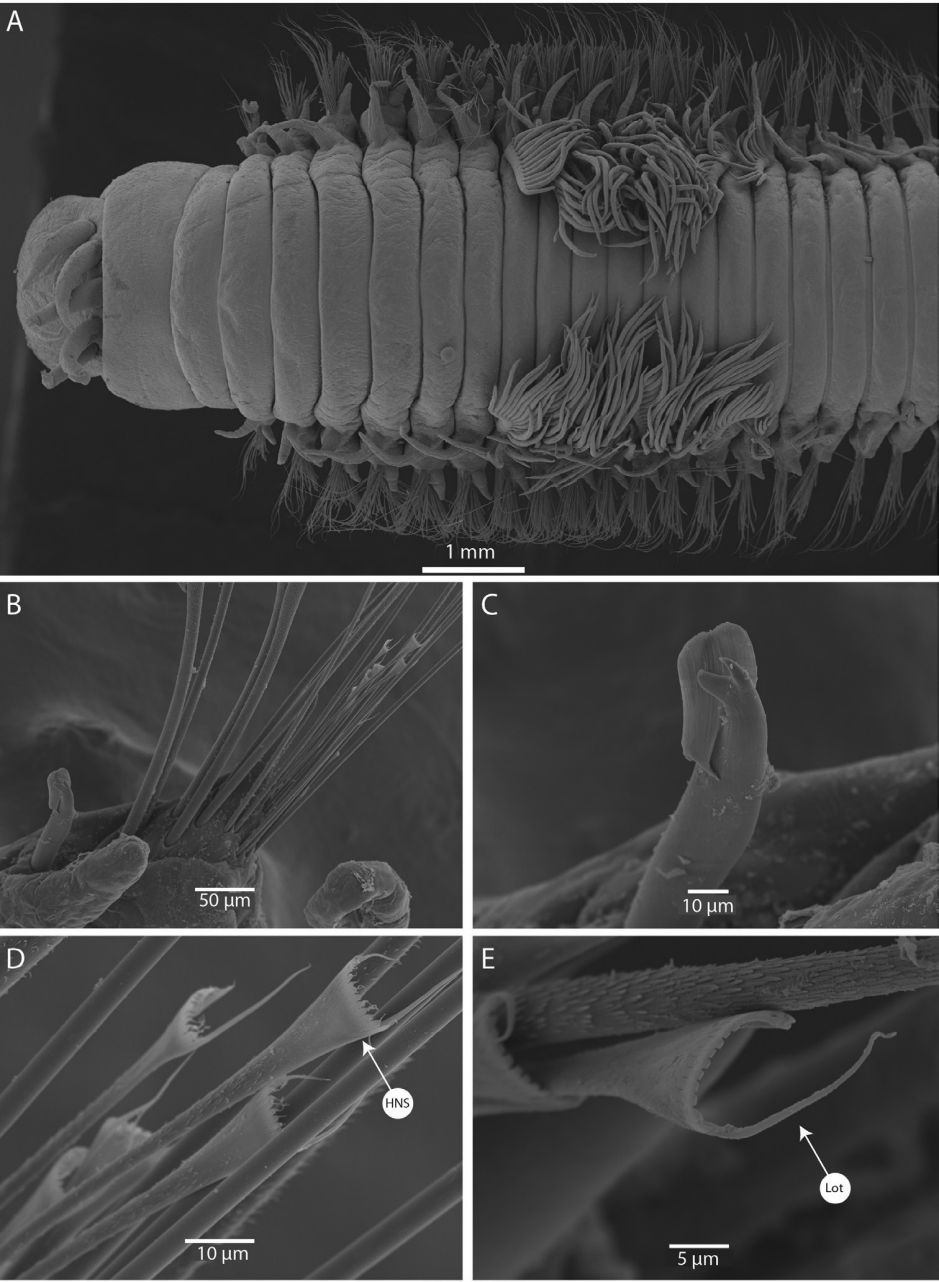
SEM images of *Marphysapapuaensis* sp. nov. paratypes AM W.53771 (**A**), AM W.53770 (**B–E**) **A** anterior end, dorsal view **B** parapodia, chaetiger 79 **C** subacicular hook, chaetiger 79 **D** pectinate chaetae, chaetiger 79 **E** pectinate chaetae, chaetiger 41. Abbreviations: HNS, heterodont-narrow-slender; Lot, Long outer tooth.

Notopodial cirri very long, slender and, tapering (Fig. 6A–C), 2–3× longer than ventral cirri in all chaetigers. Pre-chaetal lobe inconspicuous. Post-chaetal lobe digitiform in the two or three first chaetigers, triangular with tapering tip from chaetiger 4, reducing in size from chaetiger 17, almost inconspicuous from chaetiger 28 (21) (Fig. 6A–C). Ventral cirri (Fig. 6A–C) bluntly conical, with slightly expanded bases and rounded tips from chaetigers 1–22, subconical and thinner thereafter. Branchiae pectinate (Figs 5C, 6A, 7A), starting from chaetiger 7 (7) and continuing for a limited number of segments, until chaetiger 16 (14); with 8–16 long filaments.

Aciculae black with paler blunt tips, 2–4 per parapodium along the body. Supra-acicular chaetae with limbate capillaries and pectinates; capillaries present from first chaetiger to near pygidium, numbering up to 20 in anterior chaetigers. Pectinate chaetae commencing from first few chaetigers to near end, one type identified as heterodont-narrow-slender (HNS; Fig. 7B, D, E), one outer tooth very long (Fig. 7E), having nine or ten short internal teeth, each tooth prolonged by a thin filament.

Subacicular chaetae with compound falcigers and subacicular hooks (Figs 6F, G, 7B, C). Compound falcigers bidentate, with short blade and large teeth, commencing from first chaetiger to near pygidium, with more than 50 chaetae within a parapodium in anterior part, with ~ 10 chaetae in mid-body and ~ 3–5 in last chaetigers (Fig. 6F). Subacicular hooks black with pale yellow tip, commencing from anterior chaetiger 20 (24) to end, most of the body with one hook per parapodia, but some posterior chaetigers with two, subacicular hooks bidentate (Figs 6G, 7B, C).

Pygidium round and crenulated, dorsally positioned, with two pairs of tapering pygidial cirri attached at ventral edge, dorsal pair 2 (3) × length of ventral pair (Fig. 5D).

##### Etymology.

This species name refers to the type locality and geographical distribution of this species.

##### Type locality.

Papua New Guinea, Solomon Sea, New Britain.

##### Distribution.

Papua New Guinea, Solomon Sea (New Britain) and Bismarck Sea (New Ireland).

##### Habitat.

Between 250 and 1200 m, mostly found inside sunken wood.

##### Remarks.

Within the Central Indo-Pacific Realm, a single species with only compound falcigers present and branchiae restricted in a short region (group C1) has been described: *M.bernardi* Rullier, 1972 (type locality in New Caledonia). However, this species differs from *M.papuaensis* sp. nov. by the presence of a prostomium that is not bilobed, of antennae that are articulated and the absence of eyes. In contrast, *M.papuaensis* sp. nov. has smooth antennae, no eyes and a bilobed prostomium. The branchiae of *M.bernardi* are present from chaetiger 3, instead of chaetiger 7 for *M.papuaensis* sp. nov. and apparently *M.bernardi* has no pectinate chaetae, while *M.papuaensis* sp. nov. has pectinates commencing from first few chaetigers to near end. Finally, *M.bernardi* was collected in a bay from 7–8 m depth while *M.papuaensis* sp. nov. occurs only in deep sea, at 1200 m depth.

#### ﻿ Marphysa zanolae sp. nov.

Taxon classificationAnimaliaEunicidaEunicidae

3E74F0EF-84D8-5754-AB01-99910B65B639

https://zoobank.org/EAB90680-0FDD-4B21-8FB9-DD43F378A119

Figs 8 , 9

##### Material examined.

***Holotype***: MNHN-IA-2015-1519, entire, few parapodia used for molecular analysis, South Pacific Ocean, Papua New Guinea, New Ireland, CP4260, -2.9°S, 151.1°E, depth 350–847 m, April 2014. ***Paratype***: MNHN-IA-2015-1607, anterior part only, South Pacific Ocean, Papua New Guinea, New Britain, CP4266, -4.616°S, 152.25°E, depth 575–616 m, April 2014.

##### Description

(based on holotype, with variation in parentheses for paratype). Preserved specimens 197 (85 ant. part only) chaetigers, 101 mm (36 mm) long, 4.1 mm (2.8 mm) wide at chaetiger 10, excluding parapodia. Body elongated, slightly tapering at posterior end.

**Figure 8.**
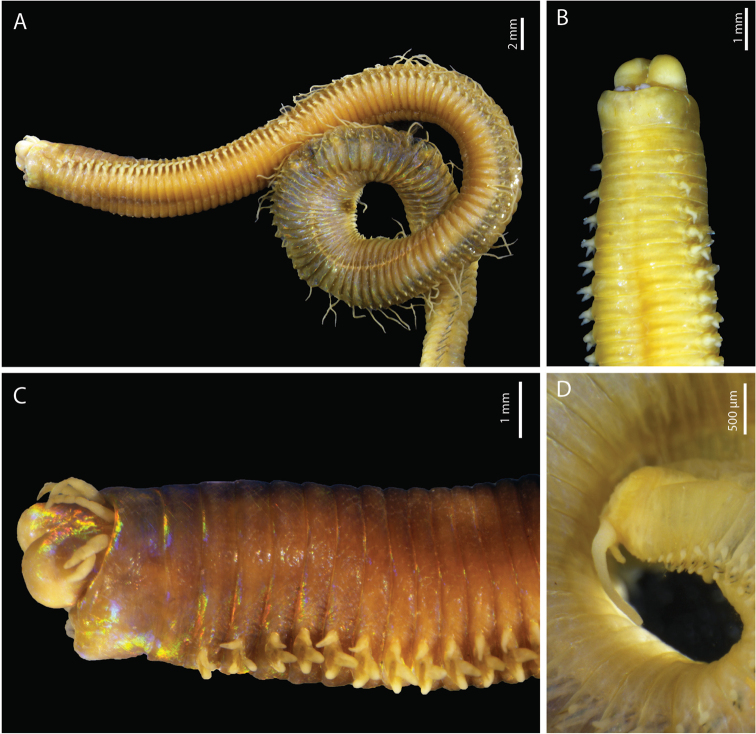
*Marphysazanolae* sp. nov. holotype MNHN-IA-2015-1519 (**A, C, D**), paratype MNHN-IA-2015-1607 (**B**): **A** anterior end, lateral view **B** anterior end, ventral view **C** anterior end lateral view **D** pygidium.

Prostomium strongly bilobed with two dorsoventrally flattened buccal lips and an anterior notch between them (Fig. 8B, C). Two palps and three antennae slender and tapering, arranged in an arc on posterior margin of prostomium. Antennae more or less smooth, of equal length, shorter than prostomium (slightly longer for PNG012), slightly longer than palps (palps very short for paratype PNG12, but probably broken) (Fig. 8C). Eyes absent. First peristomial ring approximately the same size as second one dorsally (Fig. 8C).

**Figure 9.**
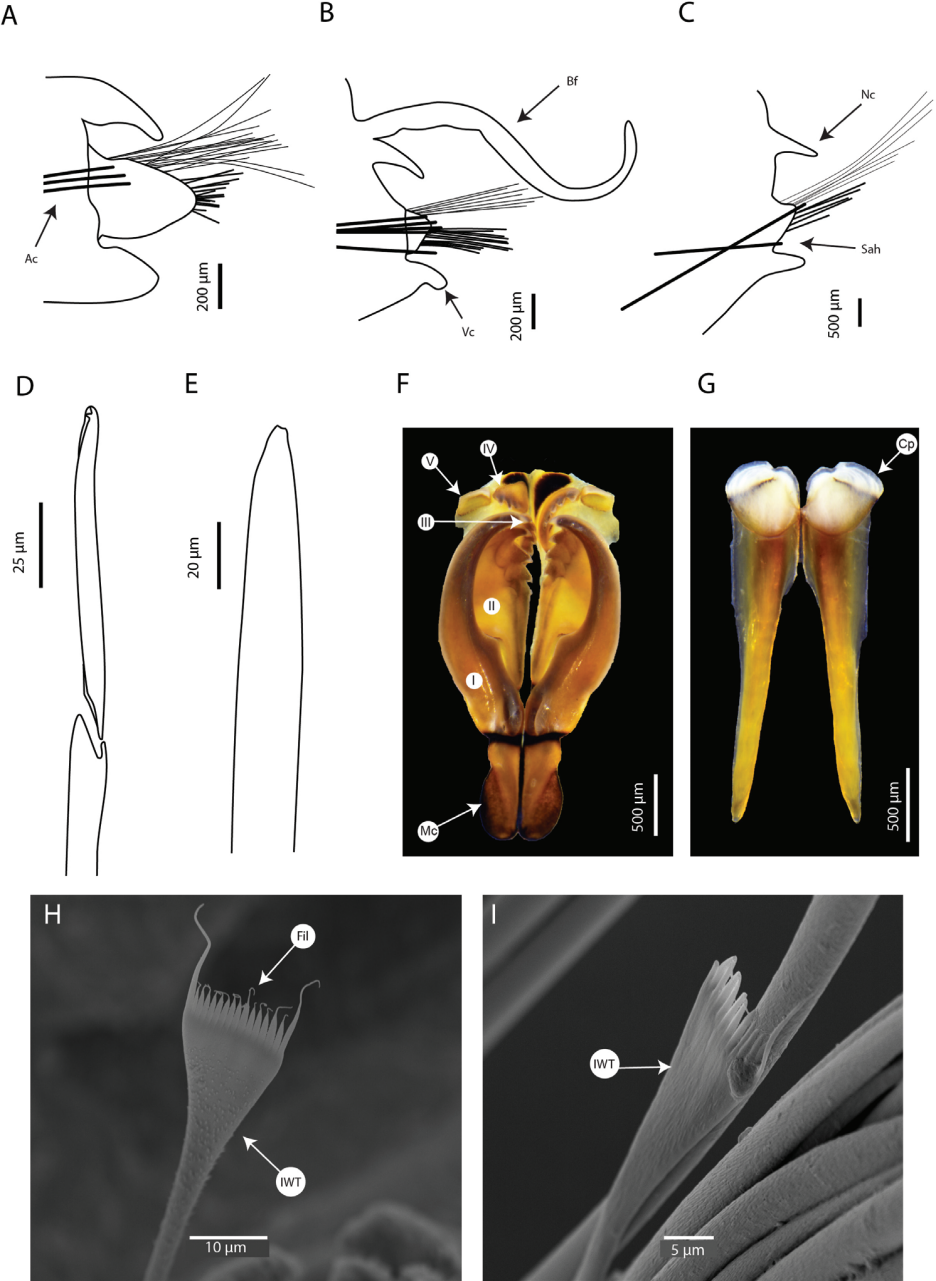
*Marphysazanolae* sp. nov. holotype MNHN-IA-2015-1519 (**A–E**), paratype MNHN-IA-2015-1607 (**F, G**): **A** parapodia from anterior body (chaetiger 8) **B** parapodia from mid-body (chaetiger 31) **C** parapodia from posterior body **D** compound falcigers from anterior chaetiger (chaetiger 12) **E** subacicular hook from mid-body (chaetiger 43) **F** maxilla, dorsal view **G** mandibles, dorsal view **H** pectinate chaeta, chaetiger 48 **I** pectinate chaeta, chaetiger 33. Abbreviations: MI to MV, maxillae I to V; Ac, aciculae; Bf, branchial filament; Cp, cutting plate; Fil, filament; IWT, isodont-wide-thick; Mc, maxillary carrier; Nc, notopodial cirri; Sah, subacicular hook; Vc, ventral cirri. **A–C** Sah and Ac are illustrated schematically to indicate position.

Maxillary apparatus yellow to golden brown, partially everted in holotype and paratype. Maxillae with carriers and four paired elements and one single one, formula as follows (Fig. 9F): MF = 1+1, 4+4, 5+0, 3+6, 1+1. MI ~ 2× longer than maxillary carrier, rectangular anteriorly, triangular posteriorly, with a pair of oval wings situated at posterolateral margins. MI forceps-like, without attachment lamellae; well-developed falcal arch. Closing system ~ 4–5× shorter than MI. MII wide, without attachment lamella, teeth triangular, recurved, and distributed in less than half of plate length. Ligament between MII and MIII absent (or not sclerotized). MIII, single, slightly shorter than right MIV, curved forming part of distal arc; with left four teeth recurved, equal-sized and triangular, two right teeth shorter and blunt, without attachment lamella. Left MIV short (half the size of right MIV) with wide, triangular base, left 2 teeth longer than right-most one; attachment lamella dark, semi-circular. Right MIV with teeth triangular, recurved, decreasing in size posteriorly; attachment lamella large, wide, best developed centrally. MV, paired, rectangular (longer than wide), with a broad cutting edge, and no clearly defined teeth (but following tradition to score as 1+1). Mandibles (Fig. 9G) yellow to golden brown, slightly shorter than MI plus carriers; cutting plates whitish, with distinct growth rings.

First two parapodia located below middle line of body wall, but gradually positioned dorsally to approximately midline in subsequent segments (Fig. 8A). Notopodial cirri with large base and slender, tapering tip from anterior to mid-body chaetigers, digitiform cirri in posterior chaetigers; same size as neuropodial cirri, but shorter than post-chaetal lobe in anterior chaetigers (Fig. 9A–C). Chaetal lobes comprising a low pre-chaetal lip and a large tongue-like post-chaetal lobe from first chaetiger to approximately chaetiger 25, almost inconspicuous thereafter. Ventral cirri bluntly conical until chaetiger 25, digitiform with bulbous base thereafter (Fig. 9A–C).

Branchiae with a long single filament (Figs 8A, 9B), commencing from chaetiger 31 (32) and continuing to mid-body (i.e., chaetiger 118 for holotype).

Aciculae black with paler blunt tips, ~ four per parapodium in anterior chaetigers, two or three per parapodium in middle chaetigers, and one or two per parapodium in posterior chaetigers. Supra-acicular chaetae with limbate capillaries and pectinates; capillaries present from first chaetiger to near pygidium, numbering up to 20 in anterior chaetigers. Pectinate chaetae commencing from first few chaetigers to near end, one type only (Fig. 9H, I), with two or three pectinate chaetae per parapodium in anterior body, up to seven from posterior chaetigers, isodont-wide-thick (IWT) having 11–20 long teeth (Fig. 9H, I).

Subacicular chaetae compound falcigers and subacicular hooks (Fig. 9D, E). Compound falcigers bidentate, with long blades and short teeth, commencing from first chaetiger to near pygidium, with more than 30 chaetae within a parapodium in anterior part, with ~ 20 chaetae in mid-body and ~ 5–7 in last chaetigers (Fig. 9D). Subacicular hooks amber to black, with much paler tip, commencing from anterior chaetiger 28 (chaetiger 30) to near end, one per parapodium in anterior and posterior parts, few chaetigers with two hooks in middle body; slightly thinner than aciculae; subacicular hooks unidentate, with blunt tip (Fig. 9E).

Pygidium round, dorsally positioned, with two pairs of tapering pygidial cirri attached at ventral edge, dorsal pair 3× length of ventral pair (Fig. 8D).

##### Etymology.

This species is dedicated to Joana Zanol for her great contributions to the knowledge of Eunicidae and *Marphysa*, and her friendship to PH.

##### Type locality.

Solomon Sea, Papua New Guinea, New Britain and New Ireland.

##### Distribution.

Only known from type locality.

##### Habitat.

Between 350 to 616 m depth, among pumice rocks, inside sunken wood.

##### Remarks.

Within the Central Indo-Pacific Realm, only one species having only compound falcigers present and branchiae present in a long region (group C2) occurs: *M.soembaensis* Augener, 1933 (type locality in Pulau Sumba, South Indonesia). However, this species differs from *M.zanolae* sp. nov. by the presence of poorly developed branchiae with two or three branchial filaments instead of well-developed branchiae with a single long filament only for *M.zanolae* sp. nov. These branchiae start from chaetiger 40 for *M.soembaensis* and from chaetiger 31 for *M.zanolae*. Moreover, *M.soembaensis* has bidentate subacicular hooks while they are unidentate for *M.zanolae* sp. nov. Finally, *M.zanolae* sp. nov. has pectinate chaetae with very long outer teeth, which are not present in *M.soembaensis*. The blade of the compound falcigers is very short for *M.soembaensis* compared to those of *M.zanolae* sp. nov. Finally, specimens of *M.soembaensis* were sampled intertidally in a bay in Indonesia while *M.zanolae* sp. nov. occurs in deep-sea environments in Papua New Guinea.

### ﻿Genetic data

**Figure 10.**
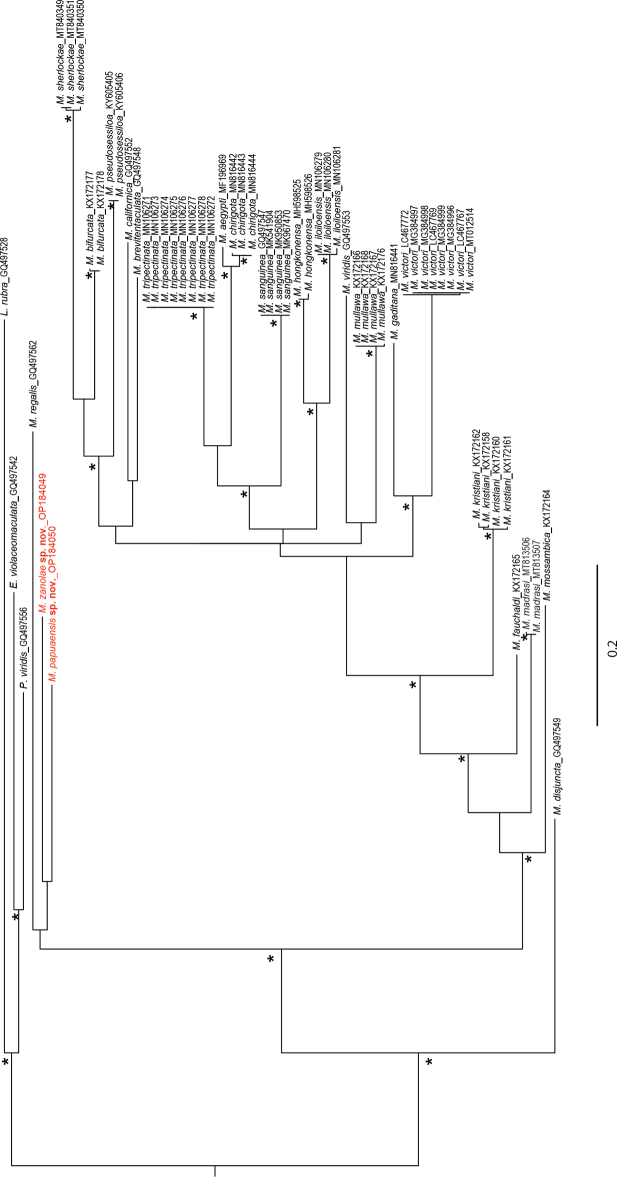
Majority-rule consensus tree of *Marphysa* species from Bayesian analysis using COI. Asterisk indicates posterior probability > 80%. Text in red indicates specimens used in this study.

COI gene was successfully sequenced and published at NCBI GenBank for two species: *M.papuaensis* sp. nov. and *M.zanolae* sp. nov. (Table 1, Fig. 10). Unfortunately, despite several attempts, sequences could not be obtained for the third species *M.banana* sp. nov. The two species *M.papuaensis* sp. nov. and *M.zanolae* sp. nov. are very different from all other species of *Marphysa* for which COI data are available and are relatively close to *M.regalis* Verrill, 1900 described from Bermuda (Fig. 10). The Pair-wise Kimura 2-parameter (K2P) between *M.papuaensis* sp. nov. and *M.zanolae* sp. nov. equal to 18.8% is relatively important and confirms the separation between these two species.

## Supplementary Material

XML Treatment for Marphysa

XML Treatment for Marphysa banana

XML Treatment for Marphysa papuaensis

XML Treatment for Marphysa zanolae
